# Supplementary material for: Impaired motor activity in a CRISPR SCA5 L253P knock-in mouse is associated with selective β-III-spectrin subcellular redistribution in the cerebellum
Source: bioRxiv. 2026 Mar 17:2026.03.14.711824. Preprint. [Version 1] doi: 10.64898/2026.03.14.711824 (PMC13015698; doi:10.64898/2026.03.14.711824)
Supplement: Supplement 1 [file media-1.pdf]

Supplemental Information

Impaired motor activity in a CRISPR SCA5 L253P knock-in mouse is associated with selective β-III-spectrin subcellular redistribution in the cerebellum

Adam W. Avery[a%#], Brennon L. O'Callaghan[b%], Matthew T. Thiel[a], Sarah A. Denha[a], Devon G. O'Callaghan[b], Emma M. Cismas[a], Jared Lamp[c], Harry T. Orr[b*#], Thomas S. Hays[d*#]

[a]Department of Chemistry, Oakland University, Rochester, MI 48309, USA
[b]Department of Laboratory Medicine and Pathology, Institute for Translational Neuroscience, University of Minnesota, Minneapolis, MN 55455, USA
[c]Integrated Mass Spectrometry Unit, College of Human Medicine, Michigan State University, Grand Rapids, MI 49503, USA
[d]Department of Genetics and Cell Biology, University of Minnesota, Minneapolis, MN 55455, USA

%Co-first authors
*Co-senior authors
#Corresponding Authors: Thomas S. Hays, Harry T. Orr and Adam W. Avery
Email: haysx001@umn.edu
Email: orrxx002@umn.edu
Email: awavery@oakland.edu

Fig. S1. L253P does not impact rotarod performance at 6 and 24 weeks.

Fig. S1.

A

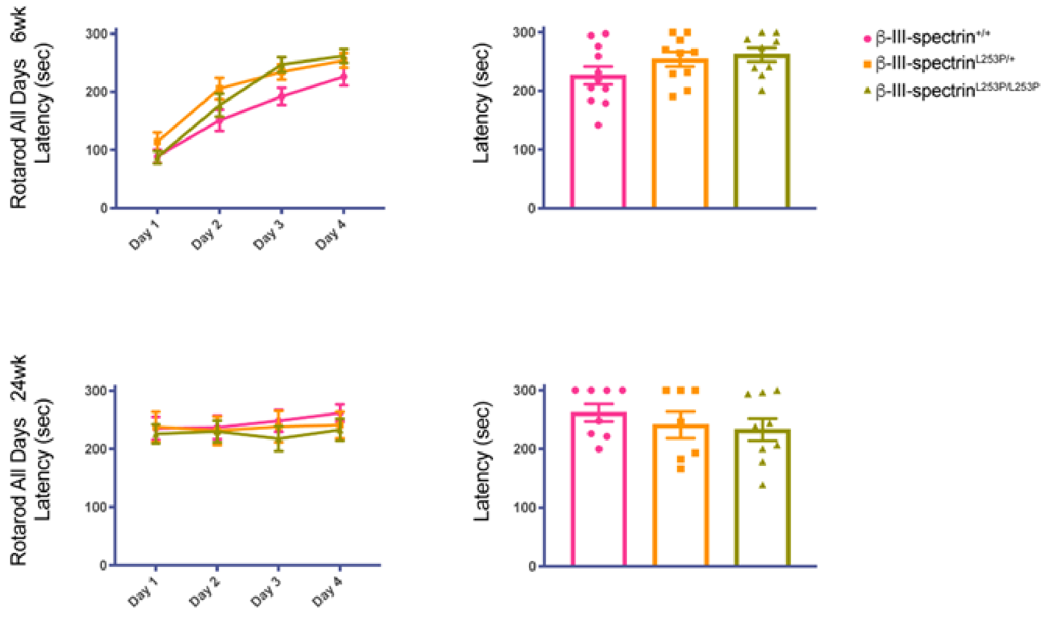

Fig. S2. Detection of β-III-spectrin in Purkinje neurons using an antibody targeting the N-terminus of β-III-spectrin.

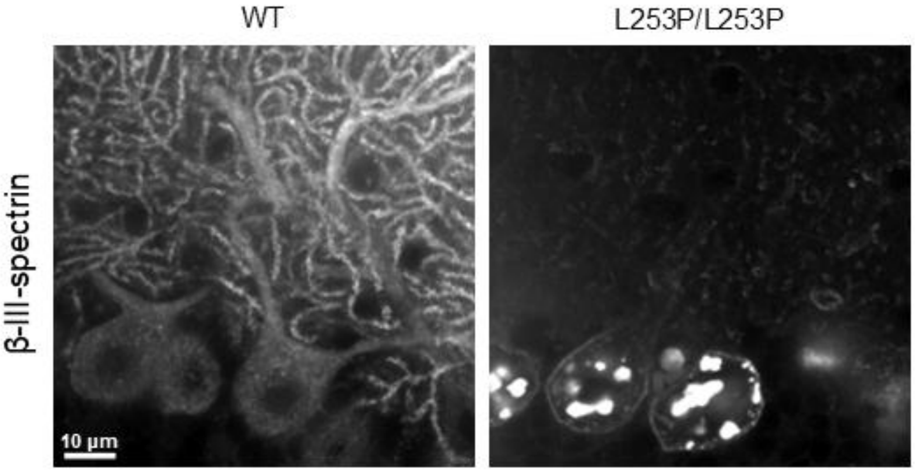

Fig. S3. Small β-III-spectrin inclusions localizing near plasma membrane.

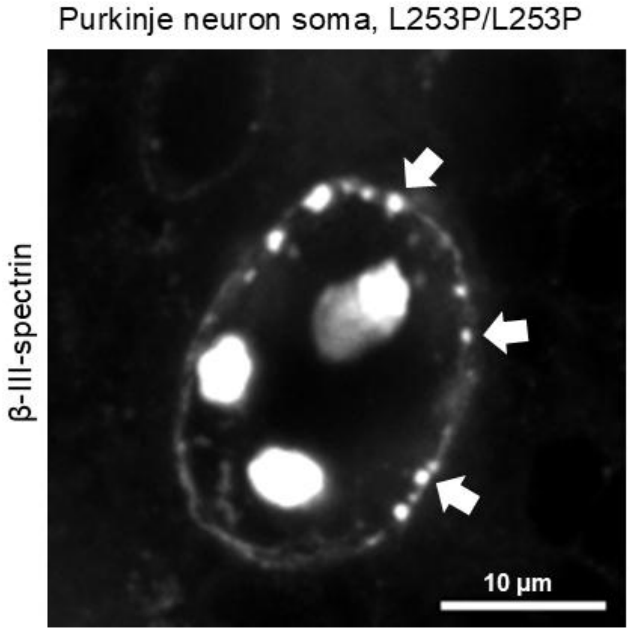

Fig. S4. Neither ankyrin-R nor EAAT4 localize to β-III-spectrin inclusions.

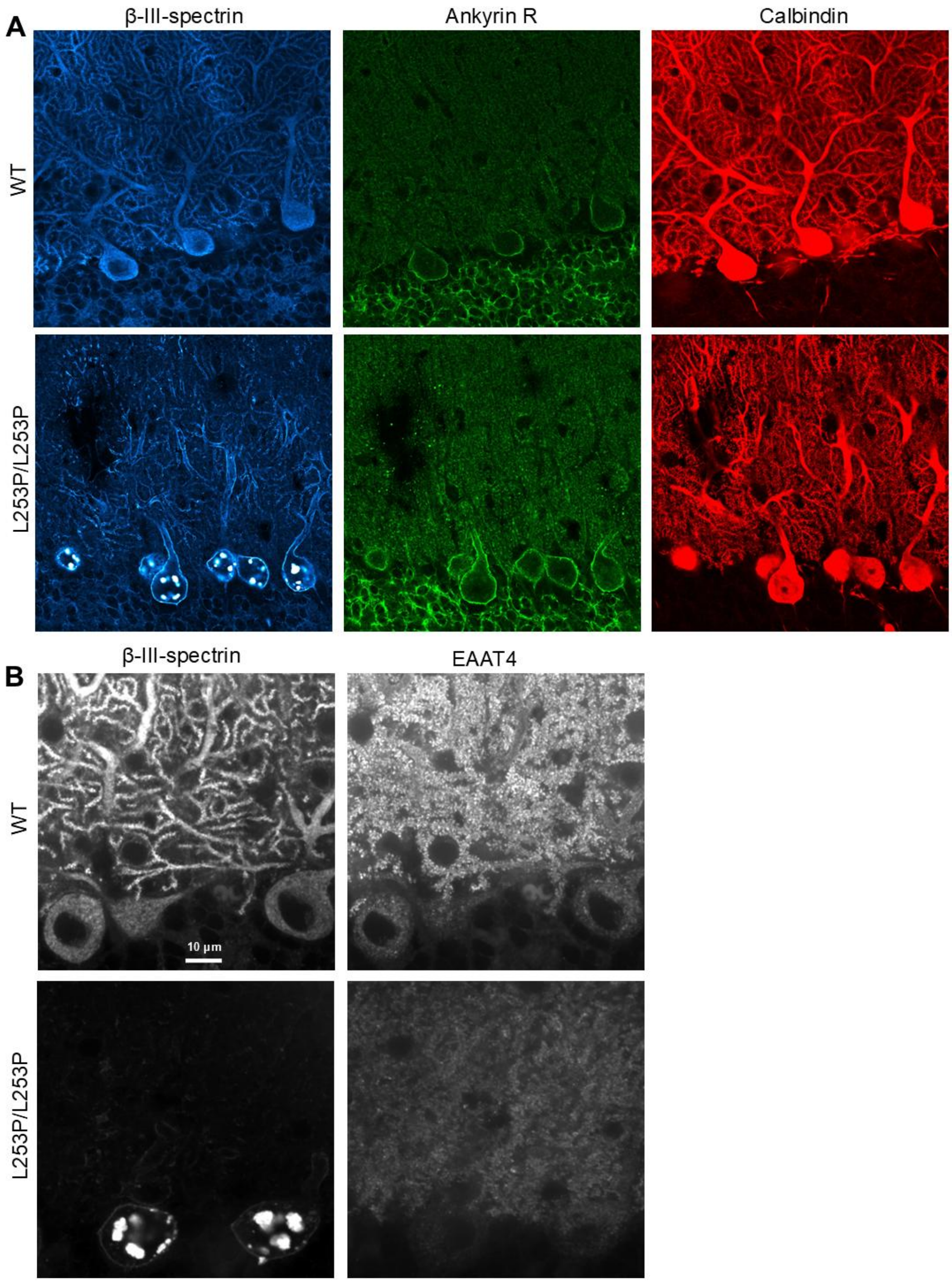

Fig. S5. A cerebellum-specific β-III-spectrin protein interactome.

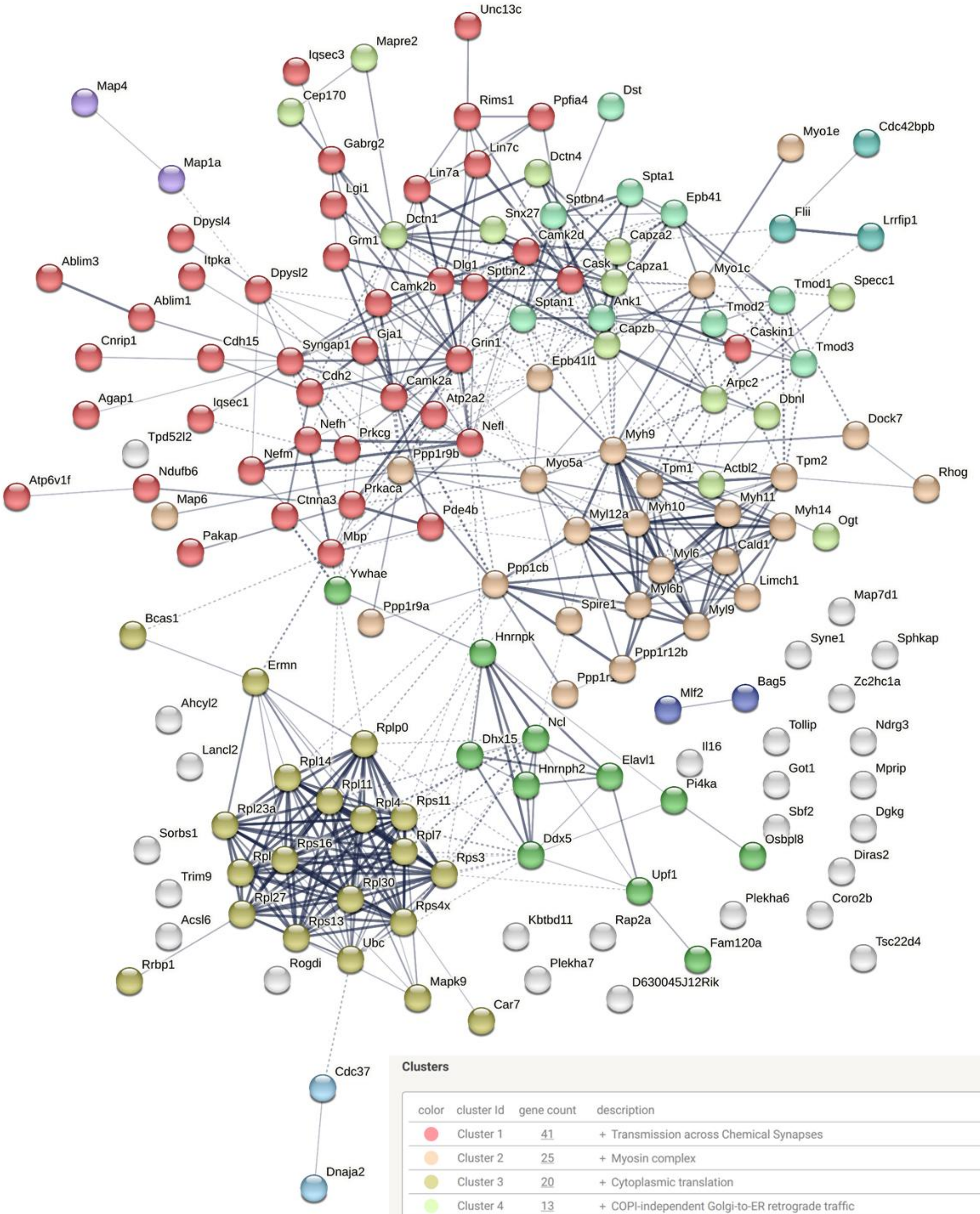

Clusters

color	cluster Id	gene count	description
	Cluster 1	41	+ Transmission across Chemical Synapses
	Cluster 2	25	+ Myosin complex
	Cluster 3	20	+ Cytoplasmic translation
	Cluster 4	13	+ COPI-independent Golgi-to-ER retrograde traffic
	Cluster 5	11	RNA recognition motif domain, and U2-type post-mRNA release spliceosomal comp...
	Cluster 6	9	+ Actin filament capping
	Cluster 7	3	Cdc42bpb, Flii, Lrrfip1
	Cluster 8	2	Regulation of HSF1-mediated heat shock response, and HSP40/DnaJ peptide-bindi...
	Cluster 9	2	Bag5, Mlf2
	Cluster 10	2	Map1a, Map4

Figure legends

Fig. S1. L253P does not impact rotarod performance at 6 and 24 weeks. Two naïve cohorts were assessed. N values for 6 week cohort: WT, 11; L253P/+, 10; L253P/L253P, 9. N values for 24 week cohort: WT, 8; L253P/+, 7; L253P/L253P, 9.

Fig. S2. Detection of β-III-spectrin in Purkinje neurons using an antibody targeting the N-terminus of β-III-spectrin. Representative confocal images of 20 week mouse Purkinje neurons.

Fig. S3. Small β-III-spectrin inclusions localizing near plasma membrane. Representative 63x confocal image from 20 week homozygous mouse. White arrows indicate small inclusions near plasma membrane.

Fig. S4. Neither ankyrin-R nor EAAT4 localize to β-III-spectrin inclusions. Representative 63x confocal images from WT and homozygous mice.

Fig. S5. A cerebellum-specific β-III-spectrin protein interactome. The interactome contains 157 proteins. Cluster analysis was performed using the STRING database.
